# Advances in the Treatment of Gastrointestinal Bleeding: Safety and Efficiency of Transnasal Endoscopy

**DOI:** 10.3390/medicines8090053

**Published:** 2021-09-14

**Authors:** Hiroyuki Abe, Kenya Kamimura, Yoshihisa Arao, Junji Kohisa, Shuji Terai

**Affiliations:** 1 Division of Gastroenterology and Hepatology, Sado General Hospital, 161 Chigusa, Sado 952-1209, Japan; hiroyukiabe@med.niigata-u.ac.jp; 2 Division of Gastroenterology and Hepatology, Graduate School of Medical and Dental Sciences, Niigata University, 1-757 Asahimachido-ri, Chuo-ku, Niigata 950-2181, Japan; y-arao@med.niigata-u.ac.jp (Y.A.); jun-g2588@angel.ocn.ne.jp (J.K.); terais@med.niigata-u.ac.jp (S.T.); 3 Department of General Medicine, Niigata University School of Medicine, 1-757 Asahimachido-ri, Chuo-ku, Niigata 951-8510, Japan

**Keywords:** upper gastrointestinal bleeding, transnasal endoscopy, esophageal stenosis

## Abstract

Acute upper gastrointestinal bleeding (UGIB) is a common disorder and a gastroenterological emergency. With the development of new techniques and devices, the survivability after gastrointestinal bleeding is improving. However, at the same time, we are facing the difficulty of severely complicated cases with various diseases. For example, while endoscopic examination with a normal diameter endoscope is essential for the diagnosis and treatment of UGIB, there are several cases in which it cannot be used. In these cases, transnasal endoscopy (TNE) may be a viable treatment option. This report reviews current hemostatic devices for endoscopic treatment and the safety and efficiency of using TNE in complicated cases. The latter will be demonstrated in a case report where TNE was employed in a patient with severe esophageal stenosis. This review summarizes the advances made in the devices used and will provide further ideas for the physician in terms of combining these devices and TNE.

## 1. Introduction

Acute upper gastrointestinal bleeding (UGIB) is a common disorder and gastroenterological emergency, with an incidence of 80–160 cases per 100,000 population [1,2]. The causes of UGIB include peptic ulcer, variceal diseases, erosive diseases, Mallory–Weiss syndrome, and other lesions, such as Dieulafoy lesions, neoplasia, hemobilia, vascular-enteric fistula, and gastric antral vascular ectasia. Peptic ulcers account for 50% of UGIB cases, whereas variceal diseases account for 5–30% and Mallory–Weiss syndrome for 5–15% [2]. The recent development of medicines and endoscopic technology has improved prognosis; however, the mortality still occurs in a small percentage of patients worldwide [3].

The first priority in UGIB is to stabilize the patient’s vitals, such as blood pressure and oxygen saturation. Infusion with fluids and/or blood is required, depending on the patient’s condition. A proton pump inhibitor or H2 blocker is also used to reduce the secretion of gastric acid; however, endoscopy is the essential procedure for definitive diagnosis and effective therapy. During initial assessment, it is important to consider endoscopic examination based on the Blatchford and Rockall scores [1]. Endoscopic examination is recommended within 24 h of initial presentation [4]. During this time frame, normal diameter endoscopes, which are for oral route use, are selected. Normal diameter endoscopes have wide operating channels to facilitate the passage of various devices. Its advantages are enhanced visibility and detection of the bleeding point by strong suction and cleaning functions. Unfortunately, there are some cases where a normal diameter endoscope cannot be used, such as in stenosis of the gastrointestinal tract.

We review current endoscopic therapy for UGIB and also the efficiency of transnasal endoscopy (TNE), including a case report in which TNE was used efficiently in a patient with benign esophageal stenosis.

## 2. Current Endoscopic Therapy by Normal Diameter Endoscope

While the number of the endoscopic examinations for the annual checkups using TNE is increasing [5], however, generally, normal diameter endoscopes are used for the screenings and therapeutic procedures for UGIB cases, with multiple benefits. Normal diameter endoscopes have wider operating channels (about 2.8–4.2 mm in diameter) compared with that of TNE (2.0–2.4 mm) [6]. The wider channel makes it possible to use various devices for hemostasis and to aspirate more contents, such as a blood clot in the stomach. In addition, water jet function and distal attachment caps to clear the bleeding point are also available in normal diameter endoscopes [7,8]. Thus, the use of a normal diameter endoscope has advantages for UGIB therapy as long as the scope can pass the gastrointestinal tract with no strictures.

There are four kinds of endoscopic procedures, namely, thermal, mechanical, injection, and spraying therapies, for hemostasis in UGIB (Table 1).

### 2.1. Thermal Therapy

The devices of thermal therapy are classified as contact type and non-contact type. In contact-type devices, the probe is put into the bleeding point, or the forceps grip the point, followed by thermal coagulation. This is a suitable method to stop bleeding from exposed blood vessels with a mucosal break, as in the case of a peptic ulcer. Conversely, in the non-contact-type therapy, argon plasma is sprayed widely onto the surface of the mucosa, where it coagulates, leading to hemostasis. This is suitable for microvascular diseases with extensive lesions, such as gastric antral vascular ectasia [9,10].

### 2.2. Mechanical Therapy

Clip and ligation therapies are the main techniques when it comes to mechanical therapy in UGIB. Clipping can close the bleeding point, such as exposed blood vessels, mechanically. Direct operation on the bleeding point by clipping leads to a better hemostatic effect [11]; however, great technical skill is precisely important to close the bleeding point. Recently, rotational and reopenable clips have been developed, compensating for any shortage of technical skill [10]. Over-the-scope clips are also useful. They are large in size and are loaded onto an attachment on the endoscope. This clip can strongly grasp wide tissues [12]. Ligation therapy is more useful for esophageal variceal rapture. Endoscopic variceal ligation uses a ligation band to ligate a variceal bleeding point along with the surrounding mucosa [13]. A suturing device is currently in development. Guided by the endoscope, this device will be able to take full-thickness bites of tissue by needle and could be used to close lesions such as ulcers [14].

### 2.3. Injection Therapy

This is the therapy in which reagents are injected into the bleeding point or surrounding mucosa. Hypertonic saline-epinephrine (HSE) solution is injected into the mucosa around the bleeding point, which causes vasoconstriction and swelling of the submucosa, leading to hemostasis or reduced hemorrhage. In spite of the efficiency of this therapy, combination therapy with other devices is recommended to prevent rebleeding [15]. Ethanol and polidocanol are also reagents used in injection therapy. Ethanol can fix tissues, including bleeding vessels. Polidocanol is injected to esophageal varices to prevent recurrence. Sclerotherapy is where histoacryl or ethanolamine is directly administered into the blood vessels. Histoacryl is injected into ruptured gastric varices [10]. Ethanolamine is used for esophageal varices.

### 2.4. Spraying Therapy

Hemostatic reagents can be sprayed through the endoscope via the operating channel. Conventionally, thrombin is the hemostatic reagent applied [16]. It is possible not to achieve a sufficient hemostatic effect with this technique as therapy is not directly applied to the bleeding point. A hemostatic powder, called TC325, is reported to be efficient in hemostatic therapy. Haddara et al. showed that in 95% of patients with peptic ulcers, initial hemostasis was achieved using TC325 [17]. TC325 is also a potential treatment for variceal hemorrhage [18]. However, there is just one randomized controlled trial about the use of TC325, and further study is required.

## 3. TNE for UGIB

**Figure 1:**
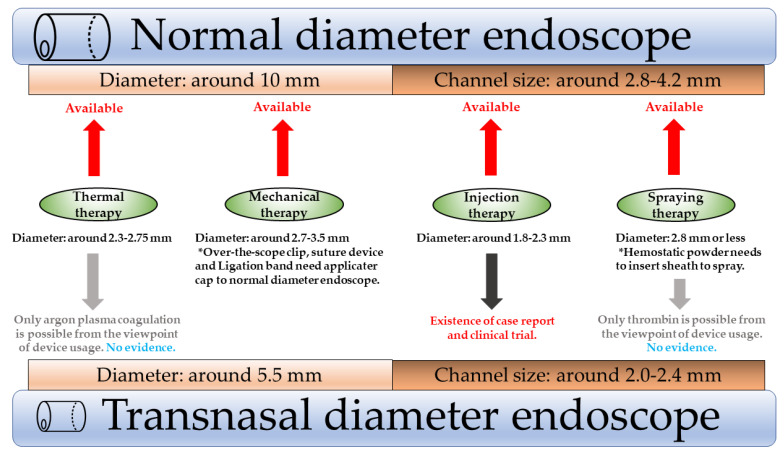
Adaption of hemostatic devices in normal and TNE.

The use of hemostatic devices with TNE is limited due to the small channel for operating and inserting devices (Figure 1). TNE has the advantage of being of thin diameter for use in UGIB cases. There are some reports on the efficacy of TNE for UGIB.

### 3.1. Current Status of TNE for UGIB

There are a few reports about the use of TNE for the treatment of UGIB. Dua et al. reported using injection therapy via TNE to treat an esophageal ulcer in a patient with trismus because of a mandible fracture [19]. In that case, HSE was injected to the vessels, leading to complete hemostasis. Mori et al. performed TNE on 210 cases of UGIB. Bleeding etiology in 97% of patients was detected via TNE, and 28% of these patients received hemostatic therapy. In 69% of treated patients, injection therapy using HSE via TNE was sufficient to achieve hemostasis [20]. Rivory et al. also reported a clinical trial using TNE to determine the etiology of hemorrhage in UGIB. They recommended TNE examination in low-risk cases based on initial clinical presentation. Doing so led to reduced deterioration of general condition and avoidance of unnecessary anesthesia [7].

Therefore, TNE should be considered in some UGIB presentations, such as low-risk cases or where there is stenosis of the gastrointestinal tract. Moreover, the utility of TNE will be increased, with the new devices currently under development.

### 3.2. Case Report: The Use of TNE in a Patient with UGIB

**Figure 2:**
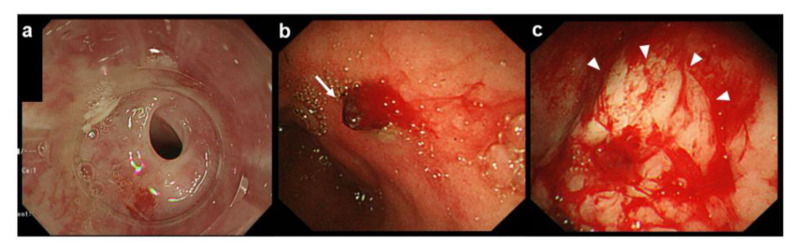
Esophageal stenosis and injection therapy by hypertonic saline-epinephrine (HSE) solution via TNE for the initial therapy. (**a**) First examination using a normal diameter scope showing esophageal stenosis; (**b**) TNE examination showing the source of the bleeding at the antrum of the stomach (arrow); (**c**) the bleeding lesion after HSE therapy. The mucosa around the lesion turns white after HSE injection (arrowheads) after successful hemostasis.

A 66-year-old man was referred to us with tarry stools and hematemesis. His heart rate and blood pressure were 106 bpm and 123/76 mmHg, respectively. Laboratory examination revealed decreased hemoglobin (10.6 g/dL) and increased blood urea nitrogen (41.6 mg/dL). Computed tomography revealed high-density stomach contents, indicating fresh bleeding. Endoscopy was attempted; however, benign esophageal stenosis by webbed stricture in the upper segment prevented insertion of the scope (GIF-Q260J, Olympus Medical, Tokyo, Japan; Figure 2a).

**Figure 3:**
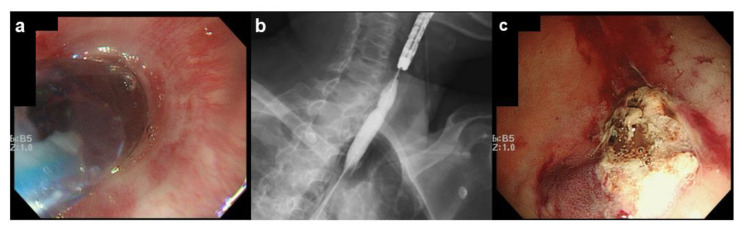
Endoscopic balloon dilation to facilitate thermal therapy at the bleeding point. (**a**) Endoscopic image during dilation; (**b**) radiograph during dilation; (**c**) endoscopic image after thermal therapy. The visible vessel has been cauterized, leading to complete hemostasis.

The scope was changed to the thinnest diameter (GIF-XP260NS, Olympus Medical, Tokyo, Japan; 5.4 mm), which could pass through the area of stenosis. A bleeding point was found at the antrum of the stomach with continuous bleeding caused by a Dieulafoy lesion and a large surface clot (Figure 2b). HSE solution was injected to the bleeding point using an injection needle through the thinnest scope to obtain the initial hemostasis and the injection achieved transient cease of the active bleeding (Figure 2c). Following this initial treatment, to achieve more effective hemostasis, the esophageal stenosis was dilated by balloon dilator to use the thermal therapy via normal diameter endoscope. The procedure went smoothly because the initial hemostasis was achieved by TNE, and the bleeding was completely stopped by cauterization (Figure 3a–c).

## 4. Conclusions

Many hemostatic applications using endoscopy have been enabled with the development of technology. Although normal diameter endoscopy has advantages in the treatment of UGIB, there are cases in which it is not a viable option. Considering the evidence and reports of TNE use in UGIB cases, as demonstrated in our case report, it appears to be an efficient alternative to normal diameter endoscopy to achieve the initial hemostasis with the cases with severe strictures in the oral side of the bleeding point. While this technique shows promise, development of hemostatic devices that can be used with TNE or thinner normal endoscopes is needed.

## Figures and Tables

**Table 1:** Current devices for endoscopic therapy in UGIB.

Type of Therapy	Devices	Advantage	Disadvantage	Adaption to Transnasal Endoscopy
Thermal therapy	Multipolar/bipolar probe Hemostatic forceps Heater probe Radiofrequency ablation probe (All are contact types)	Definite hemostasis	Risk of perforation Surrounding tissue damage by coagulation	Unavailable
	Argon plasma coagulation probe (non-contact type)	Allow coagulation to extensive lesion Coagulation of superficial tissues	Not suitable for exposed vessels	Possible from the viewpoint of device usage No evidence at transnasal endoscopy
Mechanical therapy	Conventional clip	Definite hemostasis Minimal invasion to bleeding point	Need to grasp bleeding point accurately by clip	Unavailable
	Over-the-scope clip	Close full-thickness Low risk of rebleeding	Risk of perforation Gastrointestinal obstruction	Unavailable
	Ligation band	High hemostatic effect for esophageal variceal hemorrhage	Rebleeding due to dislocation of band	Unavailable
	Suturing device	Option for refractory bleeding	Unestablished usage	Unavailable
	Stent	High clinical success rate than balloon tamponade in variceal bleeding	Need high technique	Unavailable
Injection therapy	Hypertonic saline-epinephrine solution	Easy and safety of injection Temporary stop or reduction of bleeding	Risk of rebleeding in monotherapy	Existence of case report and clinical trial
	Ethanol	High hemostatic effect for peptic ulcer	Risk of perforation	Possible from the viewpoint of device usage No evidence at transnasal endoscopy
	Ethanolamine oleate and Polidocanol	High clinical success rate for esophageal variceal bleeding	Inflow of reagent to portal vein	Possible from the viewpoint of device usage No evidence at transnasal endoscopy
	Histoacryl	High initial hemostatic rate for gastric variceal bleeding	Inflow of reagent to portal vein	Possible from the viewpoint of device usage No evidence at transnasal endoscopy
Spraying therapy	Thrombin	Non-invasive	Difficulty of hemostasis in monotherapy	Possible from the viewpoint of device usage No evidence at transnasal endoscopy
	Hemostatic powder (TC325)	High initial hemostatic rate for peptic ulcer and variceal bleeding Easy usage	Risk of rebleeding	Possible from the viewpoint of device usage No evidence at transnasal endoscopy

## Data Availability

Not applicable.
